# Evaluation of In-Person, Gluten-Free Diet Education for Children With Celiac Disease

**DOI:** 10.1097/PG9.0000000000000218

**Published:** 2022-06-21

**Authors:** Lauren Matschull, Nicole Martin, Praveen Goday, Ankur Chugh

**Affiliations:** From the * Department of Clinical Nutrition, Children’s Wisconsin, Milwaukee, WI; † Department of Pediatric Gastroenterology, Medical College of Wisconsin, Milwaukee, WI.

**Keywords:** nutrition education, patient education, registered dietitian education

## Abstract

The only treatment for celiac disease is lifelong adherence to a gluten-free diet (GFD), and the best way to achieve adherence is through education from a registered dietitian who has expertise in celiac disease. Education practices on the GFD vary across the world and are not well studied. For over 10 years, our institution has conducted in-person small group education sessions for 1–3 patients and their families. These classes are dietitian led, didactic, and discussion based. Pre- and postsurveys done for the past 5 years showed that families’ knowledge of celiac disease increased significantly and 96% of patients age 8 and above benefited from attendance. These data show that in-person, small group classes are effective for families and patients over 7 years of age. Additional study is needed to compare various models of delivering education on the GFD (especially telemedicine options), their efficacy, and barriers to delivery.

**What is Known**

-  The only treatment for celiac disease is lifelong avoidance of gluten.
-  Pediatric guidelines suggest the best way to achieve a gluten-free diet is by receiving education from a registered dietitian who has expertise in celiac disease.
-  Gluten-free diet education practices vary and are not well studied.

**What is New**

-  An in-person didactic/discussion based class, with 1–3 families, led by a registered dietitian with expertise in celiac disease, was effective at improving knowledge related to multiple aspects of the gluten-free diet.
-  The class was also effective in families who had an existing member with celiac disease.
-  Patients aged 8 and older personally benefited from attending the education class, with patients aged 6–7 also deriving some benefit.

## INTRODUCTION

Celiac disease (CeD) treatment is lifelong adherence to a gluten-free diet (GFD), which entails avoiding all foods that contain wheat, barley, and rye. This can be challenging, especially as patients age. Although pharmacologic therapies are currently being tested in clinical trials, none are currently FDA approved. The *2016 NASPGHAN Clinical Report on the Diagnosis and Treatment of Gluten-Related Disorders* emphasizes the importance of education on the GFD being led by a registered dietitian (RD) who has expertise in the treatment of CeD,^1^ as Paganizza et al^2^ found that knowledge of CeD and the GFD is strongly associated with adherence to the GFD.

Like education in other disease states, a wide range of educational methods are currently applied for CeD. However, in educating patients with CeD on the GFD, few data are available to support what method is optimal or what models are being employed. Various models include as follows: in-person, online, or blended options; via a RD with or without expertise in CeD, occurring before, during, or after a visit with a physician; using face-to-face discussions; handouts; or interactive platforms.

## METHODS

At Children’s Wisconsin, we offer an in-person, comprehensive GFD education class, led by a RD with expertise in CeD. All patients who are diagnosed with CeD are referred to our GFD education class. The class is interactive with 1–3 families present, allows for attendance by the patient and up to 2 caregivers, and ranges from 2½ to 3 hours in length. The class is offered at 2 ambulatory clinic sites (Milwaukee and Neenah) and taught by 3 different RDs. The class is taught using a combination of a PowerPoint presentation and group discussion among the families present. Families are given a binder with all materials discussed to take home. Group discussion throughout the class has proven to be very valuable, as it allows attendees to think about the information presented, ask questions, and learn from one another. The class covers what CeD is, how to read food labels, cross contact and tips for eating out, and traveling and cooking gluten-free. Nutritional risks of the GFD are also discussed, as commercially available gluten-free foods are often high in both fat and sugar and rarely enriched with vitamins and minerals. The goal is not only to teach families how to read a food label, but to teach them how to develop well-balanced gluten-free meals. Each of the RDs who teach the class use the same curriculum. We educate 80–90 families per year on the GFD across our system. Our education classes are scheduled separately from physician visits. Historically, the classes have been provided free of charge through an education grant.

At the beginning of each class, each family is given a learning assessment survey to complete. The survey asks patients/parents to disclose barriers to learning, preferred method of learning, and confidence in filling out medical forms by themselves. The families are also given an optional pre- and posteducation assessment survey to complete. Each family is asked to complete the pre-education section of the survey before the education class starting, which asks patients/parents to rank their baseline knowledge on CeD and gluten-containing foods on a Likert scale of 1–10. The pre-education survey also asks if any other family members have CeD. At the end of the class, the families are asked to complete the postsurvey (on the same form as the presurvey) and return it anonymously in a postage-paid envelope. Families rank their knowledge on the same metrics completed in the presurvey. The survey also asks if the child diagnosed with CeD attended and benefited from the class. Additionally, the survey asks how helpful they found various topics discussed (eg, definition of celiac disease, food and nonfood sources of gluten, cross contact, need for a multivitamin, and resources) on a Likert scale of 1–10 (see Survey, Supplemental Digital Content 1, http://links.lww.com/PG9/A85, for full pre- and posteducation assessment survey). A human subject research (HSR) determination form was completed for this project and submitted to the Children’s Wisconsin Institutional Review Board, but since it did not constitute HSR, an IRB submission was not required. Descriptive statistics were used to interpret the data. Paired t-tests were used for pre-/posteducation comparisons. We hypothesized that in-person education by a RD with expertise in CeD would improve knowledge of CeD and gluten-containing foods in newly diagnosed children and their families.

## RESULTS

Over a 5-year period, 462 patients and their families took our GFD education class across 2 ambulatory clinic sites (76% at Milwaukee, 24% at Neenah). Of attendees that completed the learning assessment survey, 95% (438/462) felt confident filling out medical forms all or most of the time, and only 2% (11/462) reported barriers to learning (reading, language, cognitive, and emotional). Patients ranged in age from 16 months to 18 years.

Education assessment surveys were distributed to all families who attended the class, and 106 families (23%) completed both the pre- and postsurvey (91% Milwaukee, 9% Neenah). The average knowledge (with 1 being I do not know anything and 10 being I know everything) of CeD pre-education was 5.29 and posteducation was 8.61 (*P* = 0.0001). The average knowledge of foods that contain gluten increased from 5.47 pre-education to 8.73 posteducation (*P* = 0.0001). Before attending the class, 51% of our attendees reported they received information on CeD from their healthcare professional, with additional information from the internet, family/friends, and books. All topics discussed in the class were helpful (**Table 1**), regardless of whether there was a prior family member with CeD (data not shown). Parents noted that 96% (55/57) of those aged 8 years and older benefited from attending the class (**Table 2**); 29% (2/7) of those aged 6–7 also benefitted from the class. In the 33 families (31%) who attended the class even with an existing member of the family with CeD, the pre- and posteducation knowledge of CeD and foods containing gluten increased from 5.7 to 8.7 and 6.03 to 8.97, respectively (*P* = 0.0001). Scores for any of the metrics did not differ across the 3 different RDs who teach the class. As the survey was completed anonymously, additional health literacy and demographic information on the patients/parents that completed the survey was not available.

**TABLE 1.** Evaluation of topics covered in gluten-free diet education class (n = 106)

How helpful was each topic covered in your gluten-free diet education class?	Scores										Median	IQR 1	IQR 3
	1 (%)	2 (%)	3 (%)	4 (%)	5 (%)	6 (%)	7 (%)	8 (%)	9 (%)	10 (%)			
Definition of celiac disease	0	0	0	0	2	3	7	7	16	65	10	9	10
Food Sources of gluten	0	0	0	0	1	1	5	8	22	63	10	9	10
Oats and the gluten-free diet	0	0	0	0	1	1	7	7	22	62	10	9	10
Reading labels and identifying gluten ingredients	0	0	0	0	2	1	1	7	24	65	10	9	10
Nonfood sources of gluten	0	0	0	0	1	3	1	10	20	65	10	9	10
Cross contact	0	0	0	1	0	3	1	7	14	74	10	9	10
Food and Drug Administration’s gluten-free labeling rule	0	0	0	0	1	3	4	8	16	68	10	9	10
Your child’s need for a multivitamin	0	0	0	0	1	2	3	5	14	75	10	9	10
Allowable grains, seeds, and starches	0	0	0	2	1	2	5	5	18	67	10	9	10
Reliable sources of information	0	0	0	0	0	1	3	9	20	67	10	9	10
Gluten-free resources (stores, books, websites)	0	0	0	0	1	3	2	7	17	70	10	9	10

1= not helpful at all; 10= extremely helpful.

IQR = interquartile range.

**TABLE 2.** Age when child benefited from attending gluten-free diet education class (n = 69)

Age of child (years)	Attended class	Benefited from class
3	2/69 (3%)	0/2 (0%)
4	2/69 (3%)	0/2 (0%)
5	1/69 (1%)	0/1 (0%)
6	4/69 (6%)	1/2 (50%)
7	3/69 (4%)	1/2 (50%)
8	4/69 (6%)	3/4 (75%)
9	2/69 (3%)	2/2 (100%)
10	6/69 (9%)	5/6 (83%)
11	5/69 (7%)	5/5 (100%)
12	13/69 (19%)	13/13 (100%)
13	6/69 (9%)	6/6 (100%)
14	7/69 (10%)	7/7 (100%)
15	3/69 (4%)	3/3 (100%)
16	10/69 (14%)	10/10 (100%)
17	0/69 (0%)	N/A
18	1/69 (1%)	1/1 (100%)

It was based on a total of 69 children that attended the class with their parents; data points are missing from two 6-year-olds and one 7-year-old who attended the class on whether they benefited from the class.

## DISCUSSION

Our education assessment surveys show that our format of an in-person, small group GFD education class, with both didactics and discussion, led by RDs with expertise in CeD, was successful in increasing attendee knowledge of both CeD and the GFD. Families with an existing family member with CeD also benefitted, which has not been shown in previous studies. Whether the patient had a prior family member with CeD did not change the results. Starting a GFD is a significant lifestyle change for both children and their parents, so children, especially those 8 years and older, should attend a GFD education class with their parents.

Our study has some limitations. Our education assessment survey was optional, and only 23% were returned. This creates the possibility of a selection bias, as patients who completed the survey may have done so due to a more positive experience. Only 2% of attendees reported barriers to learning, which mitigates the risk of this population struggling with the class or not responding to the survey as a result of poor health literacy. Adherence to the GFD and long-term knowledge retention were not tracked. One of our survey questions “did your child benefit from the class” is very broad and does not provide insight on why children over 7 years benefited. Additionally, we used a 10-point Likert scale for several survey questions, and some data suggest that Likert scales greater than 7 may be less accurate.^3^

Other studies have looked at different formats and the efficacy of GFD education in both pediatric and adult patients. Elsahoryi et al^4^ studied education sessions for parents with children who have CeD. These included a RD and were held as in-person, large group sessions with 20 parents in each group. The education was provided over 2 different sessions that were spaced 2 weeks apart at a medical center in Jordan. Their study of 40 parents who completed both education sessions found that parent knowledge level regarding CeD and GFD increased when comparing pre/postsurveys. This improved parents’ attitudes and practices and consequently improved patients’ self-reported adherence to the GFD. Connan et al^5^ aimed to develop and test the usability of an interactive e-learning module targeted at educating patients and their caregivers on the implementation of the GFD for children with both CeD and type 1 diabetes. The e-learning module was tested in 2 iterative cycles on a total of 18 patients and 15 caregivers with changes being made to the module between cycles based on participant feedback. The module was effective in increasing short-term knowledge retention on the GFD, and both patients and caregivers rated the module highly enjoyable. In adults, Sainsbury et al^6^ conducted a randomized controlled trial to assess the effectiveness of online education in improving GFD adherence and GFD knowledge. The intervention group of 50 adults completed 6 weekly online modules, whereas the control group did not receive any formal education on the GFD. Their interactive online education program improved both long-term GFD knowledge and adherence compared with those in the control group who did not receive the education.

While our celiac education model differs from other published models in format and design, we continue to see that the best way to achieve a GFD is by receiving education from a RD who has expertise in CeD. Our in-person education classes were successful in increasing attendee knowledge of both CeD and the GFD, but they are lengthy and allow for only 2 caregivers to attend with the patient due to space limitations. Our education classes also require a significant amount of face-to-face time with a RD. Due to unique rules in each state regarding reimbursement for RD charges, variability exists in whether patients are charged to attend GFD education. A 2005 survey found that most patients must pay for GFD education, which can range from $60 to $295/hour, and can be a barrier to patients receiving education.^7^ In a different survey of 1612 US adults with CeD, Green et al^8^ found that 66% of patients were referred to a RD; however, the majority, 88%, claimed they obtained the bulk of their information about CeD from celiac patient support groups.

Online education has been used for several chronic diseases as a tool to increase knowledge and improve compliance.^9^ Online education has many advantages, as it allows for additional caregivers to participate in the education and allows families to learn at their own pace and time. It also could increase patient access to GFD education, decrease costs, and provide a safe option during periods of pandemics such as COVID-19. However, just as in traditional education, everyone has different learning styles, and many learn better in-person with the ability to ask questions as they arise and learn from the people around them. Thus, since the GFD is the only treatment for CeD, it would be beneficial to start comparing different education models across different programs and within programs themselves (assuming they can provide multiple education formats) to look at the differences in short- and long-term knowledge retention as well as adherence to the GFD, to ultimately deliver the best, most cost-effective care for pediatric patients with CeD.

## ACKNOWLEDGMENTS

The authors would like to thank Hope Quilling for her contributions.

## Supplementary Material


