# Supplementary material for: Evaluation of In-Person, Gluten-Free Diet Education for Children With Celiac Disease
Source: JPGN Rep. 2022 Jun 21;3(3):e218. doi: 10.1097/PG9.0000000000000218 (PMC10158309; doi:10.1097/PG9.0000000000000218)
Supplement: Supplementary file 1 [file pg9-3-e218-s001.pdf]

Celiac Education Assessment Survey
Part 1
(to be completed before class)

The purpose of this survey is to determine the needs of families recently diagnosed with celiac disease and then to determine how well I met those needs in this education session.

This survey is meant to be anonymous. I have provided a stamped envelope for mailing your completed survey back. I do not need your name, your child's name or any other identifiers on your survey. All I ask is for your honest opinion to help me improve!

1. Where did you find the information/knowledge you currently have on Celiac disease? Check all that apply.
   - ☐ Internet
   - ☐ Books
   - ☐ Family/Friends
   - ☐ Healthcare Professional
   - ☐ Other (explain)

   ____

2. Do any other family members have celiac disease? ☐ Yes ☐ No

   The following questions can be answered by circling your response on a scale of 1 to 10 where:
   (1= *I don't know anything*, 10= *I know everything*)

3. I would say that my knowledge of celiac disease is:
   1 2 3 4 5 6 7 8 9 10

4. I would say that my knowledge of what foods contain gluten is:
   1 2 3 4 5 6 7 8 9 10

****

Part 2
(to be completed after class)

The following questions can be answered by circling your response on a scale of 1 to 10 where:
(1= *I don't know anything*, 10= *I know everything*)

5. After the class, I would say that my knowledge of celiac disease is:
   1 2 3 4 5 6 7 8 9 10

6. After the class, I would say that my knowledge of what foods contain gluten is:
   1 2 3 4 5 6 7 8 9 10

7. Regarding the various topics covered in the class, how helpful did you find each topic?
   The following questions can be answered by circling your response on a scale of 1 to 10 where:
   (1= *Not helpful at all* 10= *Extremely helpful*)

Definition of Celiac Disease	1	2	3	4	5	6	7	8	9	10
Food sources of gluten	1	2	3	4	5	6	7	8	9	10
Oats and the gluten free diet	1	2	3	4	5	6	7	8	9	10
Reading labels and identifying gluten ingredients	1	2	3	4	5	6	7	8	9	10
Non food sources of gluten	1	2	3	4	5	6	7	8	9	10
Cross contact	1	2	3	4	5	6	7	8	9	10
FDA's Gluten Free Labeling Rule	1	2	3	4	5	6	7	8	9	10
Your child's need for a multivitamin	1	2	3	4	5	6	7	8	9	10
Allowable grains, seeds, and starches	1	2	3	4	5	6	7	8	9	10
Reliable sources of information	1	2	3	4	5	6	7	8	9	10
Gluten free resources (stores, books, websites)	1	2	3	4	5	6	7	8	9	10

8. What was the most helpful topic that we discussed in class?

   ____

9. What was least useful topic that we discussed in class?

   ____

10. Was the Celiac education class worth your time?
    (1= not worth my time, 10=worth every second)
    1 2 3 4 5 6 7 8 9 10

11. Did your child attend the Celiac education class? ☐ Yes ☐ No
    If yes, how old is your child? ___________________
    Did your child benefit from the class? ☐ Yes ☐ No

12. Who taught your education class? ______________________________________

13. Were you part of a group or individual class? ____________________________

14. Anything else that you would like to comment on:

    ____

Thank you for taking the time to complete this survey!!
